# *NLRC3* is a potential prognostic biomarker that is correlated with immune cell infiltration in lung adenocarcinoma

**DOI:** 10.1038/s41598-022-23979-z

**Published:** 2023-02-20

**Authors:** Yingchen Zhuo, Xueqian Li, Weiyi Feng

**Affiliations:** 1 Department of Pharmacy, The First Affiliated Hospital of Xi’an Jiaotong University, No. 277 Yanta West Road, Xi’an, 710061 Shaanxi China; 2 Department of Endocrinology, The Second Affiliated Hospital of Xi’an Jiaotong University, Xi’an, 710043 Shaanxi China

**Keywords:** Cancer, Cancer microenvironment, Lung cancer, Tumour biomarkers, Tumour immunology

## Abstract

The NLR family CARD domain containing 3 (*NLRC3*) gene has been reported to have a crucial effect on immunity, inflammation, and tumorigenesis. However, the clinical relevance of *NLRC3* in lung adenocarcinoma (LUAD) remains unclear. This study analyzed both RNA sequencing data and corresponding clinical outcomes obtained from public databases to identify (i) *NLRC3* as a tumor suppressor in LUAD and (ii) its predictive value for the likelihood of patient responsiveness to immunotherapy. The results showed that *NLRC3* expression was reduced in LUAD and was lower in advanced-stage tumors. Additionally, reduced *NLRC3* expression was correlated with worse patient prognosis. The protein level of NLRC3 was also observed to have prognostic significance. Moreover, downregulation of *NLRC3* was found to suppress the chemotaxis and infiltration of antitumor lymphocyte subpopulations as well as natural killer cells. Mechanistic analysis indicated that *NLRC3* may be involved in immune infiltration by regulating chemokines and their receptors in LUAD. Furthermore, *NLRC3* functions as a molecular switch in macrophages, whereby it mediates the polarization of M1 macrophages. Patients with high *NLRC3* expression were also found to exhibit a more promising response to immunotherapy. In conclusion, *NLRC3* could serve as a potential prognostic biomarker for LUAD, help predict the immunotherapeutic response of patients, and guide personalized strategies for the treatment of LUAD.

## Introduction

Lung adenocarcinoma (LUAD) is the most common type of non-small cell lung cancer (NSCLC), and most affected patients are in an advanced, inoperable stage^1^. Chemotherapy is often the primary treatment for “driver gene-negative” LUAD. Recent rapid developments in immune checkpoint blockade (ICB) have yielded remarkable clinical gains. Unfortunately, only 19% of randomly selected patients with NSCLC respond to PD-1 blockade, and treatment response predictions for individual patients remain challenging^2–4^. As a result, identifying useful biomarkers to enable patient selection and avoid toxicity in nonresponders is urgently needed.

The NLR family CARD domain containing 3 (*NLRC3*) gene, located on chromosome 16p13.3, encodes the NOD-like receptor family member NLRC3. NLRC3 generally has various regulatory functions in immunity and inflammation^5,6^. Recent studies have shown that NLRC3 is dysregulated across many cancers and implicated in their progression. For example, NLRC3 prevents colorectal cancer growth by suppressing the PI3K-mTOR signaling pathway and may suppress hepatocellular carcinoma (HCC) progression by promoting CD8^+^ T-cell infiltration^7,8^. However, information available on the expression, regulation, and clinical significance of NLRC3 in LUAD remains lacking.

This study used bioinformatics to investigate the potential functions of *NLRC3* in LUAD. The available data indicate that *NLRC3* is a potent prognostic biomarker that is correlated with immune infiltration in LUAD and that its intrinsic expression levels in LUAD tumors may influence the outcomes of therapies involving ICB.

## Results

### *NLRC3* gene expression in LUAD

**Figure 1:**
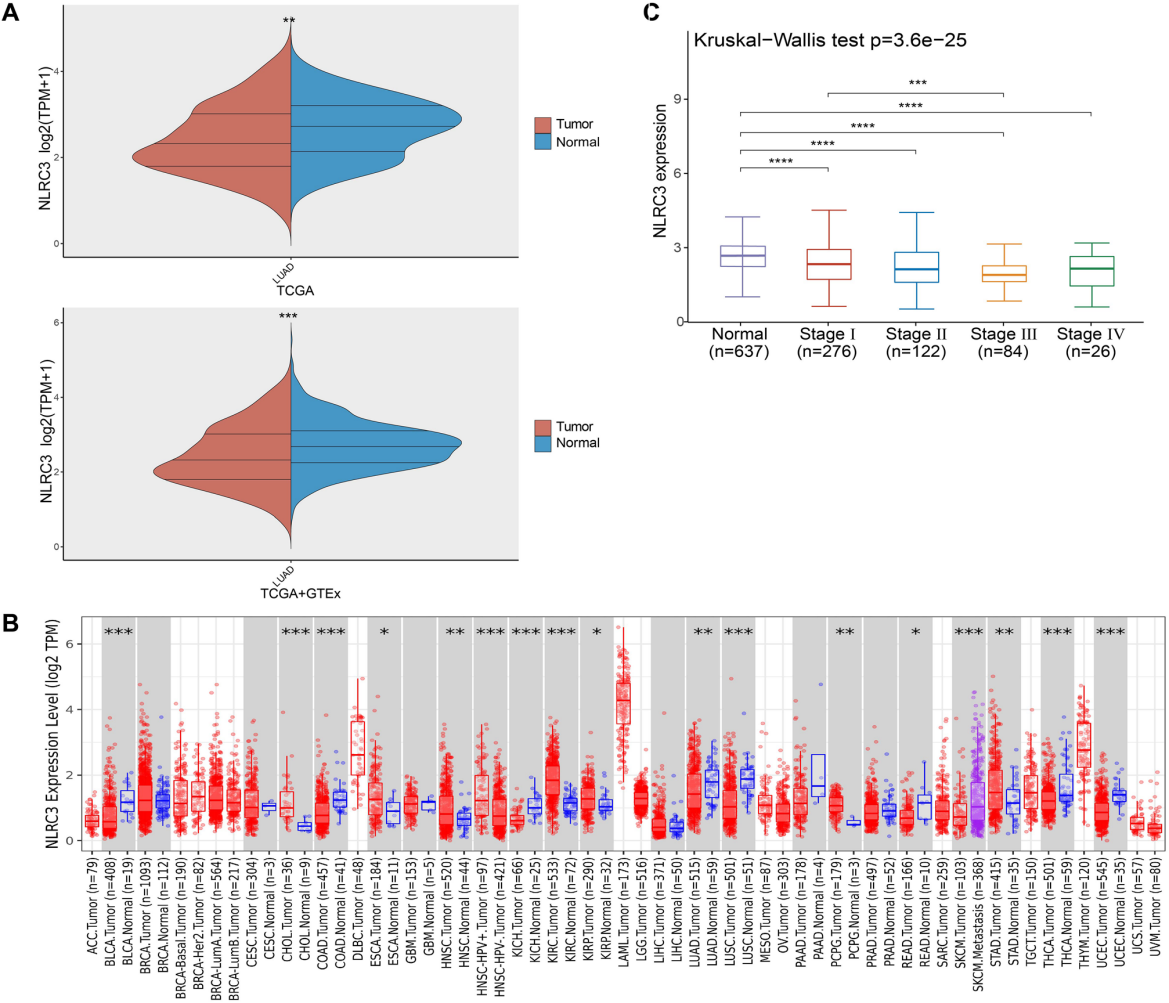
Different mRNA expression levels of NLR family CARD domain containing 3 (*NLRC3*) in lung adenocarcinoma (LUAD) and normal tissues. (**A**) *NLRC3* mRNA expression levels in LUAD and normal tissues. Analysis was performed using the software R version 4.0.3. (**B**) The mRNA expression levels of *NLRC3* in different cancer types were explored by TIMER2.0. (**C**) Association between *NLRC3* expression and tumor stage. Analysis was performed using the software R version 4.0.3. *P < 0.05, **P < 0.01, ***P < 0.001.

To evaluate *NLRC3* expression in LUAD tissue, we analyzed the RNA sequencing profiles from 513 patient samples, which revealed that *NLRC3* had markedly lower mRNA expression levels in LUAD than in normal tissues (Fig. 1A). Consistent with data from the TIMER2.0 database, *NLRC3* expression was reduced in LUAD (Fig. 1B). The *NLRC3* expression level was also correlated with tumor stage, and its level was significantly higher in early-stage tumors (stage I) than in advanced-stage tumors (stage III) (Fig. 1C). These results suggest that *NLRC3* downregulation in LUAD is correlated with cancer progression.

### *NLRC3* was associated with better survival and was an independent prognostic factor for LUAD

**Figure 2:**
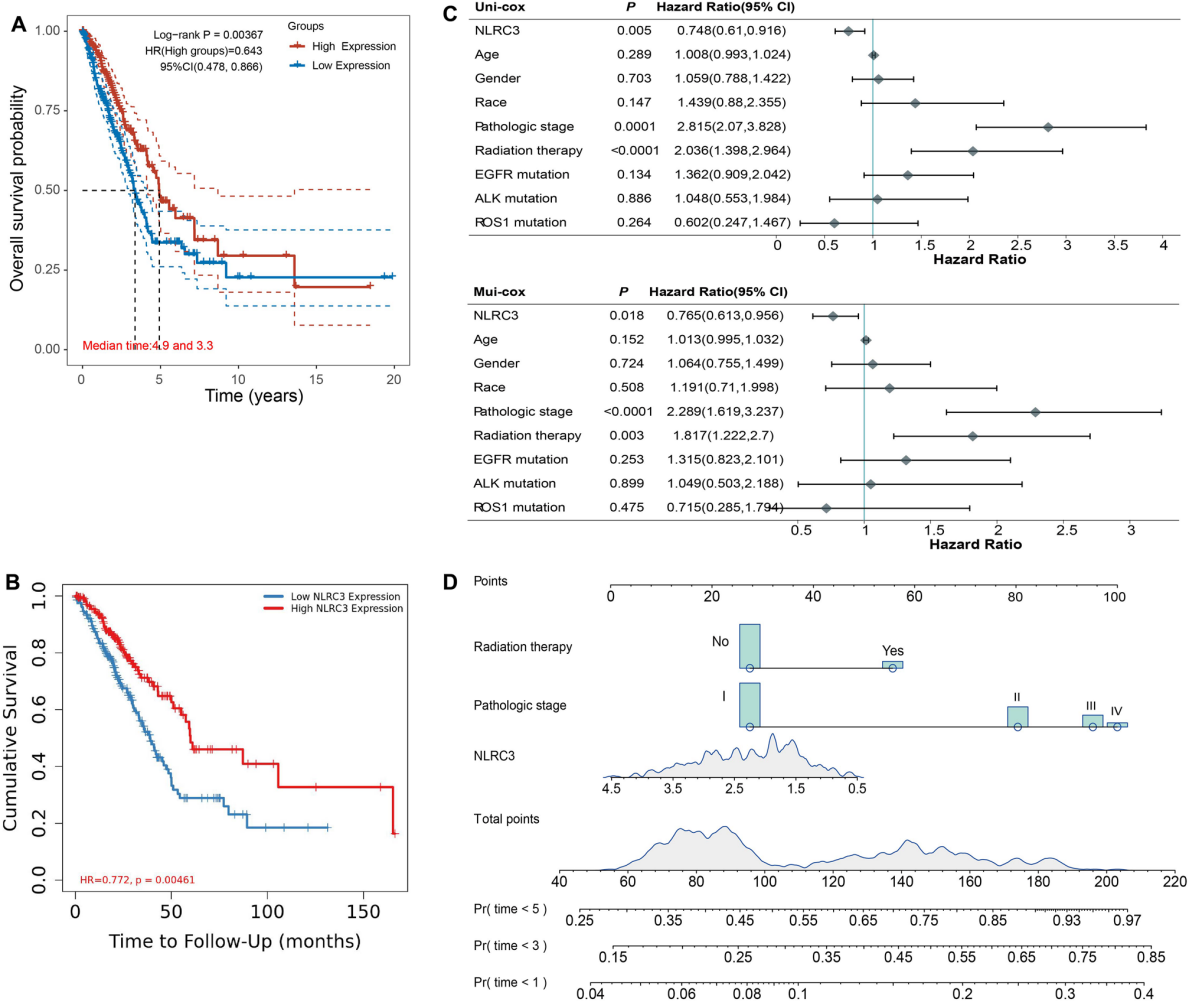
*NLRC3* was associated with improved survival and was an independent prognostic factor in LUAD. (**A**) Overall survival (OS) of patients with LUAD grouped according to the median *NLRC3* expression level. Analysis was performed using the software R version 4.0.3. (**B**) OS of patients with LUAD grouped according to the median *NLRC3* expression level from TIMER2.0. (**C**) Results from univariable and multivariable regression analyses of prognostic factors for OS. Analysis was performed using the software R version 4.1.1. (**D**) Nomogram for predicting 1-, 3-, and 5-year survival rates in patients with LUAD. Analysis was performed using the software R version 4.1.1.

High *NLRC3* expression in LUAD was associated with improved patient OS (overall survival; the length of time a patient lives following the initiation of treatment) (HR = 0.643, *P* = 0.00367; Fig. 2A). Similar results were also obtained for the validation cohort from the TIMER2.0 database (HR = 0.772, *P* = 0.00461; Fig. 2B). The independent prognostic value of nine factors in patients with LUAD was further assessed by conducting univariable and multivariable Cox regression analyses (Fig. 2C). Univariable analysis showed that *NLRC3* (HR = 0.748, *P* = 0.005), pathologic stage (HR = 2.815, *P* = 0.0001), and radiation therapy (HR = 2.036, *P* < 0.0001) were associated with prognosis, with *NLRC3* (HR = 0.765, *P* = 0.018), pathologic stage (HR = 2.289, *P* < 0.0001) and radiation therapy (HR = 1.817, *P* = 0.003) remaining significant prognostic factors for OS in multivariable analysis. *NLRC3* expression was therefore an independent prognostic factor for patients with LUAD. Moreover, a nomogram was constructed to predict the 1-, 3-, and 5-year survival rates of patients with LUAD according to *NLRC3* expression, pathologic stage and radiation therapy (Fig. 2D).

### NLRC3 protein expression and prognosis in LUAD

**Figure 3:**
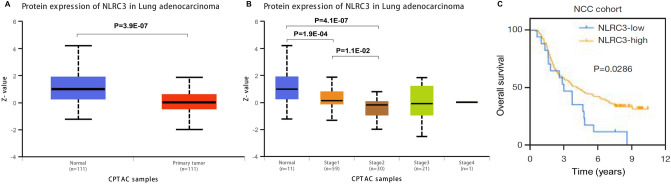
NLRC3 protein expression and prognosis in LUAD. (**A**) The protein expression level of NLRC3 in LUAD and normal tissues using the UALCAN database. (**B**) The association between NLRC3 protein expression and tumor stage in LUAD using the UALCAN database. (**C**) The survival curves for OS of patients with high/low NLRC3 expression from the National Cancer Center of China (NCC) LUAD cohorts. This figure and the corresponding result were originally published in Wang et al.^9^. Reused with permission from AME Publishing Company.

The protein expression level of NLRC3 was further analyzed using the UALCAN (the University of Alabama at Birmingham Cancer Data Analysis Portal) omics-based web resource. The results showed that NLRC3 expression was significantly downregulated in LUAD tissues compared with normal tissues (Fig. 3A). The NLRC3 expression level was also correlated with tumor stage (Fig. 3B). Moreover, LUAD patients with high expression of NLRC3 exhibited a longer OS (*P* = 0.0286; Fig. 3C)^9^.

### Correlation between *NLRC3* and the tumor microenvironment (TME)

**Figure 4:**
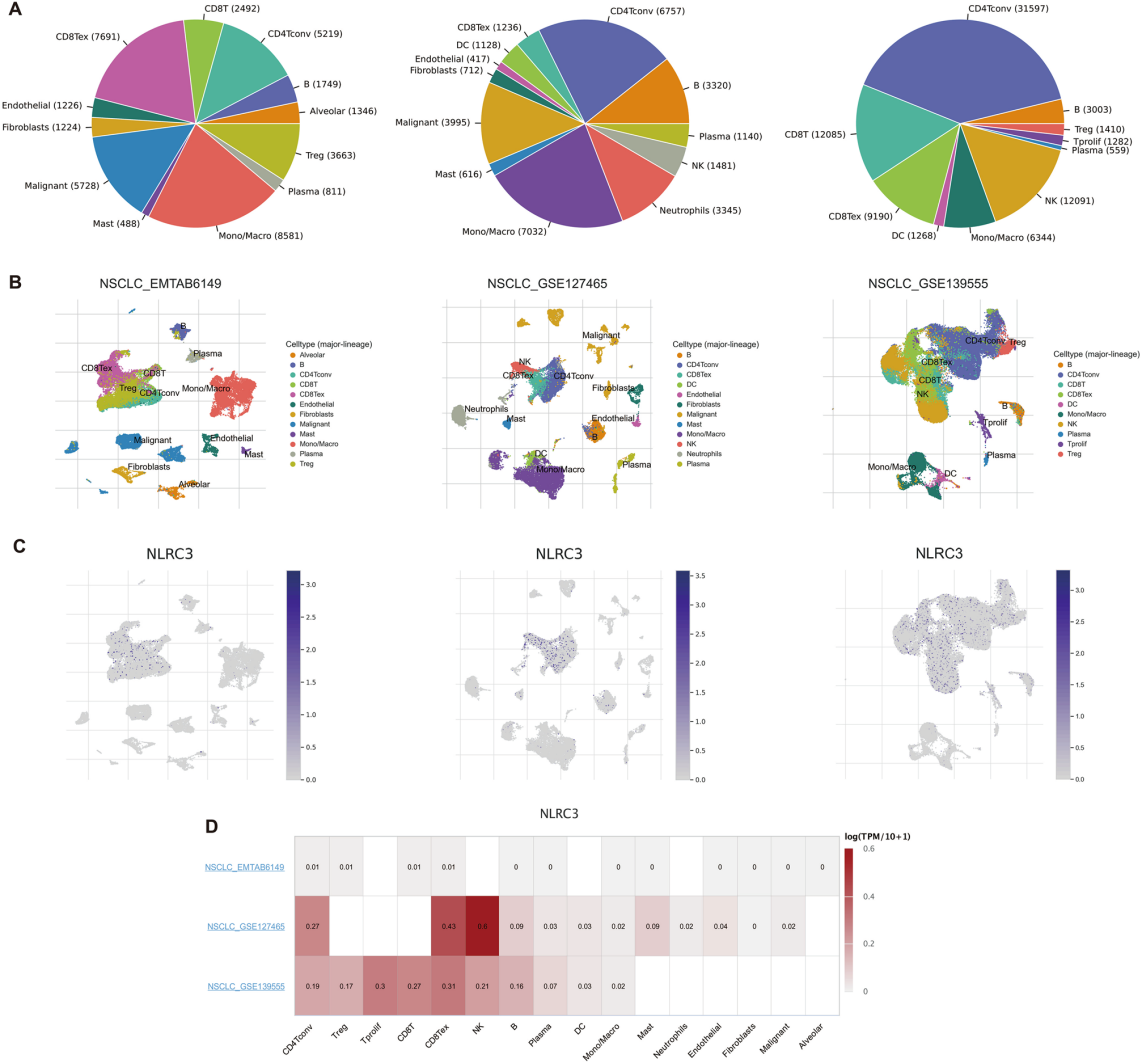
Correlation between *NLRC3* expression and the tumor microenvironment using the TISCH database. (**A**) The cell types and their distributions in the NSCLC_EMTAB6149, NSCLC_GSE127465, and NSCLC_GSE139555 datasets. (**B**,**C**) Distribution of *NLRC3* across different cells in the NSCLC_EMTAB6149, NSCLC_GSE127465, and NSCLC_GSE139555 datasets. (**D**) The heatmap displays the value of *NLRC3* expression in different cells from different databases.

We used three single-cell RNA-seq datasets (NSCLC_EMTAB6149, NSCLC_GSE127465, and NSCLC_GSE139555) obtained from the TISCH database containing data for non-small cell lung cancer to analyze the expression of *NLRC3* in tumor microenvironment (TME)-related cells. As shown in Fig. 4A, immune cells comprised an important component of the TME. *NLRC3* was mainly expressed in immune cells (Fig. 4B,C). Additional analysis showed that the *NLRC3* expression level was markedly different between distinct cell types, with the highest levels measured in CD4^+^ T cells, CD8^+^ T cells, and natural killer (NK) cells (Fig. 4D).

### *NLRC3* is associated with the immune process or immune-related pathways in LUAD

**Figure 5:**
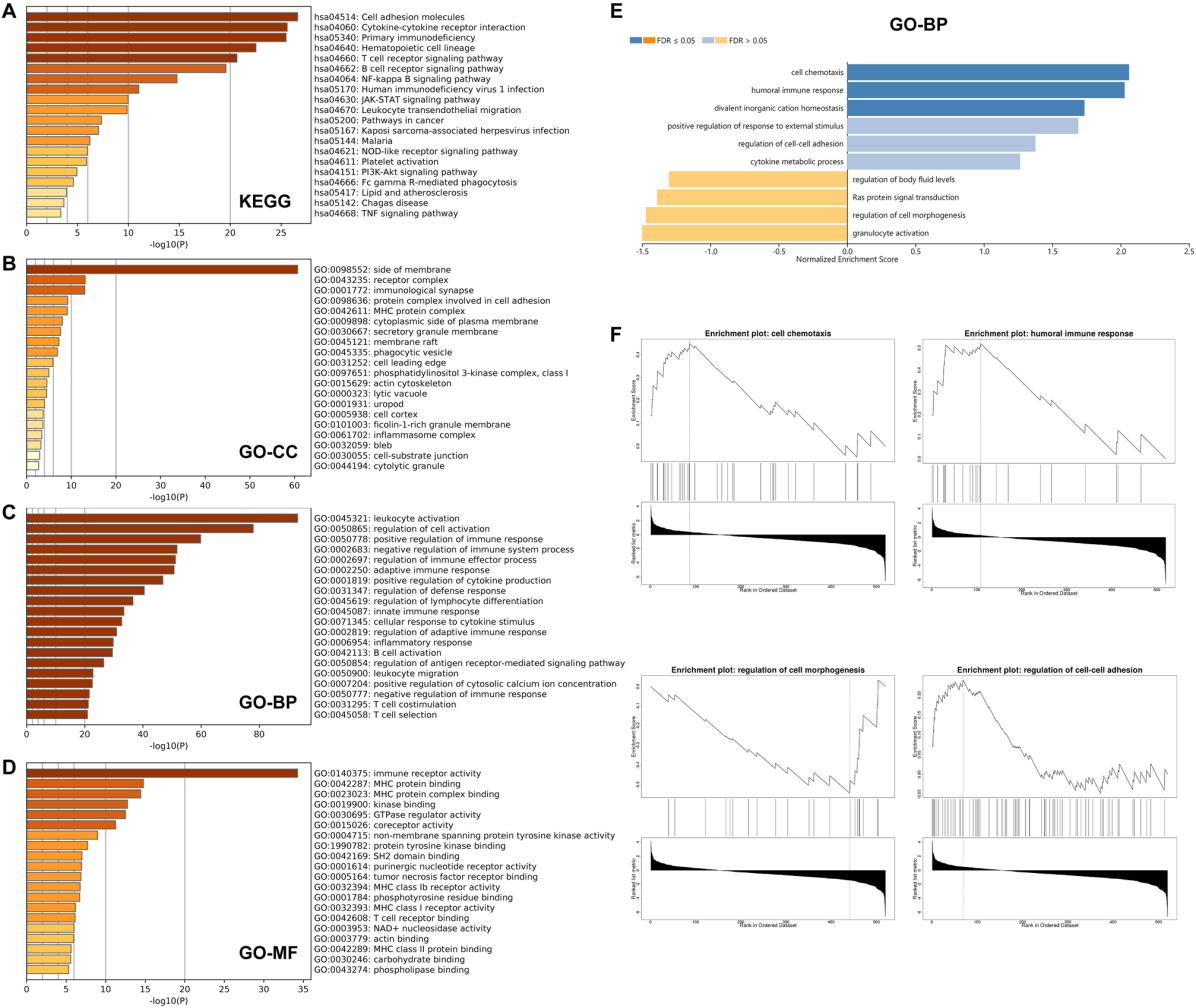
Enrichment analysis of *NLRC3* in LUAD using the Metascape or WebGestalt database. (**A**–**D**) Kyoto Encyclopedia of Genes and Genomes and Gene Ontology pathways of the 696 genes most positively associated with *NLRC3*. (**E**,**F**) Gene set enrichment analysis results for *NLRC3*.

The mechanisms underlying how *NLRC3* affects LUAD were explored by analyzing the correlations between *NLRC3* and other genes using TCGA data. The 696 genes most positively associated with *NLRC3* (Cor > 0.5, *P* < 0.05) were selected for the enrichment analysis, which was conducted using Metascape and WebGestalt. The results from Kyoto Encyclopedia of Genes and Genomes pathway analysis suggested that *NLRC3* was mostly correlated with cell adhesion molecules (Fig. 5A). Gene Ontology (GO) enrichment analysis revealed that *NLRC3* was positively correlated with immune processes or immune-related pathways, including lymphocyte activation (Fig. 5B–D). Significant GO annotation through gene set enrichment analysis showed that *NLRC3* coexpressed genes participate in cell chemotaxis, while cell morphogenesis process regulation was inhibited (Fig. 5E,F).

### Correlation between *NLRC3* expression and immune cell infiltration

**Figure 6:**
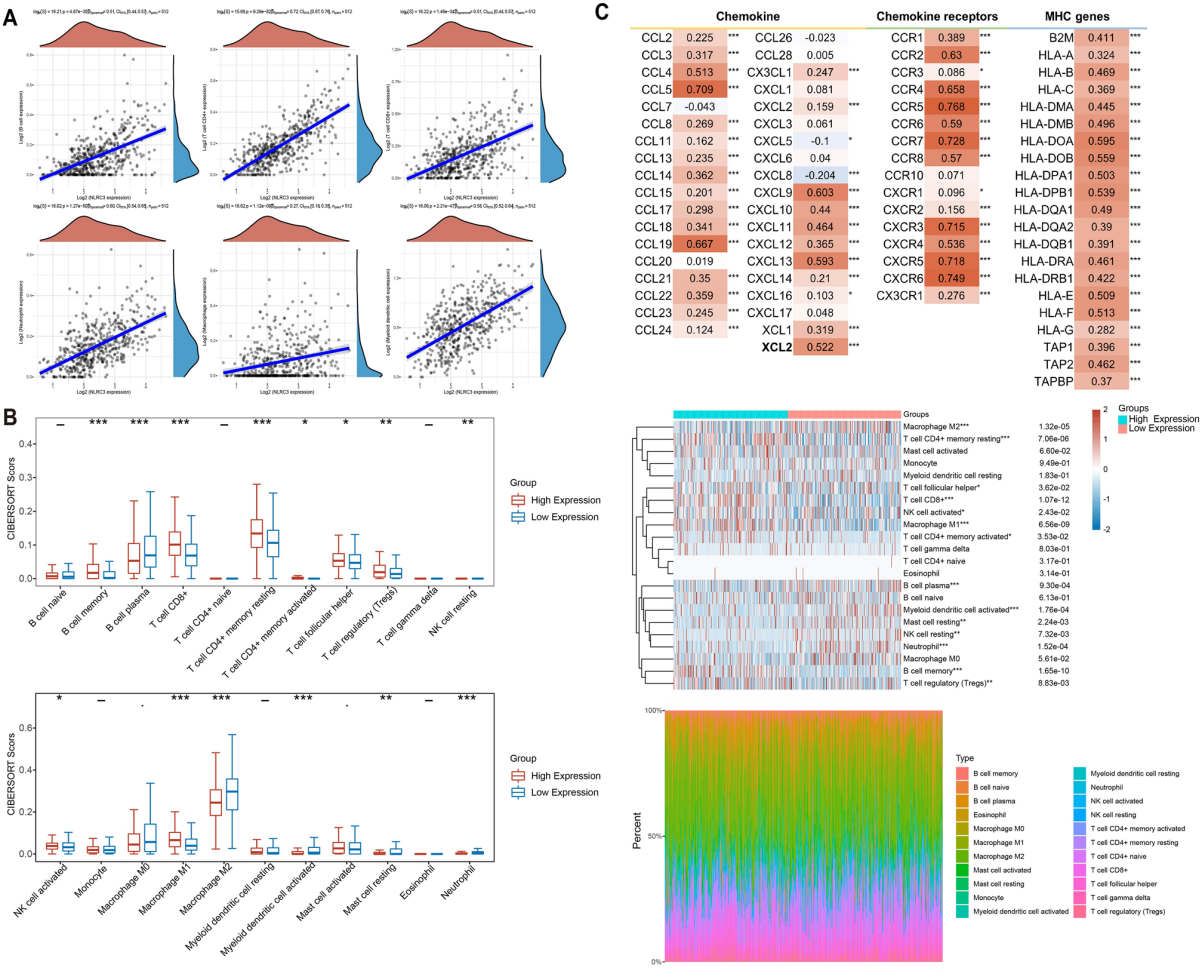
Correlation between *NLRC3* expression and immune cell infiltration. (**A**) Positive correlations between *NLRC3* expression and the infiltration levels of six immune cell types in patients with LUAD. Analysis was performed using the software R version 4.0.3. (**B**) Differences in the proportions of infiltrating immune cells between the high- and low-*NLRC3*-expression groups. Analysis was performed using the software R version 4.0.3. (**C**) The correlations among *NLRC3*, chemokines, and chemokine receptor expression were analyzed using the TISIDB database. *P < 0.05, **P < 0.01, ***P < 0.001.

We first used the TIMER2.0 algorithm to calculate the correlations between *NLRC3* expression and the infiltration levels of six immune cells. Figure 6A shows that *NLRC3* expression had moderate and strong correlations with immune cell infiltration in patients with LUAD (B cells, Spearman’s ρ = 0.51, *P* < 0.001; CD4^+^ T cells, Spearman’s ρ = 0.72, *P* < 0.001; CD8^+^ T cells, Spearman’s ρ = 0.51, *P* < 0.001; neutrophils, Spearman’s ρ = 0.60, *P* < 0.001; macrophages, Spearman’s ρ = 0.27, *P* < 0.001; dendritic cells, Spearman’s ρ = 0.58, *P* < 0.001).

We next investigated the differences in immune cell infiltration between patients with LUAD with high and low *NLRC3* expression. The proportions of 22 tumor immune cell types are shown in Fig. 6B. Patients with LUAD with high *NLRC3* expression had significantly higher proportions of memory B cells, CD8^+^ T cells, memory CD4^+^ T cells, follicular T helper (T_H_) cells, regulatory T cells, activated NK cells, and M1 macrophages (*P* < 0.05). However, the numbers of plasma B cells, resting NK cells, M2 macrophages, activated dendritic cells, resting mast cells, and neutrophils were observed to be significantly increased in patients with low *NLRC3* expression (*P* < 0.05).

We also analyzed how *NLRC3* affects the chemotaxis of immune cells in LUAD (Fig. 6C). The results indicated that *NLRC3* was positively coexpressed with chemokines and their receptors. *NLRC3* showed moderate and strong correlations with *CCL5*, *CXCL9*, *CXCL10*, *CXCL11*, *CXCR3*, and *CCR5*, resulting in an increased recruitment of NK cells, T_H_1 cells, and CD8^+^ T cells in tumor tissues. *CXCR4* and *CXCL12* showed moderate correlations with *NLRC3*, thereby facilitating the recruitment of B cells. The MHC complex was also positively correlated with *NLRC3*, indicating that *NLRC3* promotes antigen presentation.

Together, these observations suggest that *NLRC3* promotes the infiltration of antitumor lymphocyte subpopulations and functions as a molecular switch in macrophages, mediating the polarization of M1 macrophages.

### LUAD patients with high *NLRC3* expression may benefit from ICB treatment

**Figure 7:**
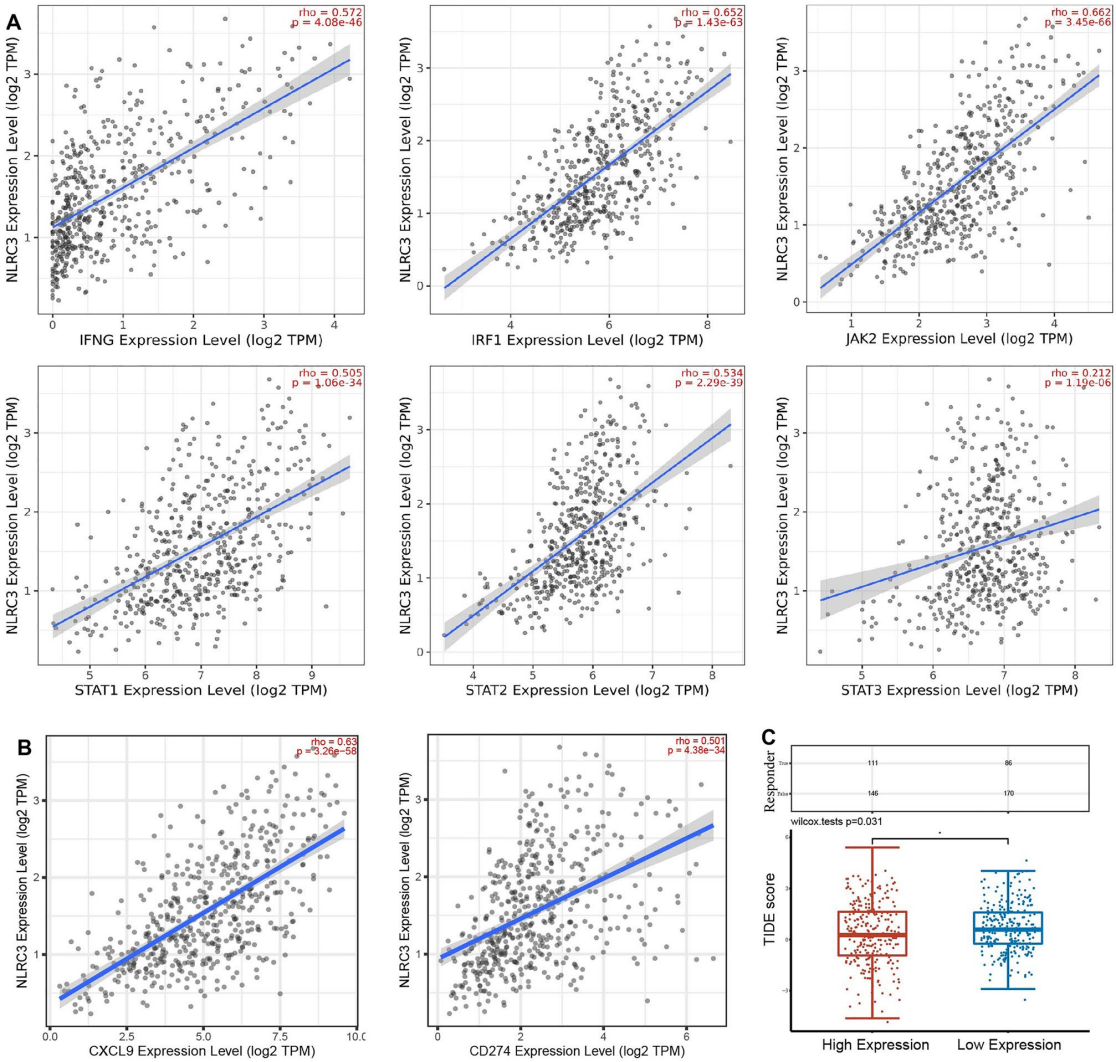
Patients with LUAD with high *NLRC3* expression may benefit from immune checkpoint blockade (ICB) treatment. (**A**) Correlations between *NLRC3* and six downstream interferon-γ signaling molecules using the TIMER2.0 database. (**B**) Spearman’s coefficient for the correlation between *NLRC3* and *CXCL9*/*CD274* using the TIMER2.0 database. (**C**) Differential ICB responses in patients with LUAD grouped according to the median *NLRC3* expression level. Analysis was performed using the software R version 4.0.3. *P < 0.05.

To demonstrate the role of *NLRC3* in ICB treatment, we first explored the relationships between *NLRC3* and the interferon-γ signaling gene, the latter of which is known to determine patient responses to clinical ICB therapy. The results indicated that downstream interferon-γ signaling molecules, including *IFNG*, *JAK2*, *STAT1*, *STAT2*, *STAT3*, and *IRF1*, were positively correlated with *NLRC3* (Fig. 7A). We then explored the relationships between *NLRC3* and *CXCL9*/*CD274*. As shown in Fig. 7B, *NLRC3* was positively correlated with *CXCL9* and *CD274* (Spearman’s ρ = 0.63, *P* < 0.001; Spearman’s ρ = 0.501, *P* < 0.001). We further predicted the clinical response to ICB of patients with LUAD with high and low *NLRC3* expression using the TIDE algorithm. A lower tumor TIDE score was associated with a better ICB response and patient survival under anti-PD-1 and anti-CTLA-4 therapies. Figure 7C shows that patients with high *NLRC3* expression exhibit more promising immunotherapy responses (*P* = 0.031).

## Discussion

This study demonstrated that *NLRC3* acts as a tumor suppressor in LUAD. *NLRC3* expression was reduced in LUAD, and it was expressed at lower levels in advanced-stage tumors, indicating that *NLRC3* downregulation promotes tumor progression. Importantly, reduced *NLRC3* expression was correlated with worse patient prognosis. The protein level of NLRC3 was also observed to have prognostic significance. Subsequent univariable and multivariable analyses verified that the *NLRC3* expression level is an independent prognostic indicator for LUAD.

Previous studies have confirmed that *NLRC3* prevents colorectal cancer growth by suppressing the PI3K-mTOR signaling pathway and that it may suppress HCC progression by promoting CD8^+^ T-cell infiltration^7,8^. Our study revealed a significant correlation between the expression of *NLRC3* and the tumor immune microenvironment of LUAD. Single-cell sequencing of NSCLC was used to explore the TME, and the results showed that *NLRC3* was mainly expressed in immune cell types. Functional analysis showed that *NLRC3* was closely related to immune processes and immune-related pathways. High and low *NLRC3* expression was related to different immune profiles in LUAD; high *NLRC3* expression indicated a high level of immune cell infiltration, particularly by antitumor lymphocyte subpopulations, activated NK cells, and M1 macrophages. However, with low *NLRC3* expression, the levels of immune suppressor cells (such as M2 macrophages) were observed to be significantly elevated in patients.

Mechanistic analysis showed that *NLRC3* may be involved in immune infiltration by regulating chemokines and their receptors in LUAD. Chemokines serve as extracellular signals, and chemokine receptors detect chemokine gradients to guide different immune cell subsets into tumors^10,11^. Positive correlations were found between *NLRC3* and chemokines (and their receptors) in LUAD, especially for *CCL5*, *CXCL9*, *CXCL10*, *CXCL11*, *CXCR3*, and *CCR5*. Previous studies have determined that the cooperation between CCL5 and CXCL9 and their mutual receptor CXCR3 is important for inducing T-cell infiltration in immunoreactive and immunoresponsive tumors^12^. CXCL9, CXCL10, CXCL11, and their shared receptor CXCR3 play a key role in driving T_H_1 and CD8^+^ T cells into the TME^11^. *NLRC3* also promotes antigen presentation. We therefore propose that upregulation of NLRC3 increases the infiltration of effector immune cells (NK cells and CD8^+^ T cells), thereby changing a “cold tumor” into a “hot tumor” and driving tumor regression.

Immune checkpoint blockade represents a groundbreaking recent advance in the treatment of cancers, having shown considerable efficacy across many cancer types^13^. Unfortunately, only 19% of randomly selected patients with NSCLC have been shown to respond to PD-1 blockade^2–4^. Thus, there has remained the important challenge of identifying biomarkers that can reliably predict which patients will benefit most from ICB treatments^14^. Enriched T_H_1-type chemokines, effector immune cells, and functional antigen presenting cells that reside in the TME are reported to be favorable for clinical responses to ICBs^11^. The presence of activated effector T cells is a primary factor known to predict responses to ICB^14^. The expression of *CD274*, *CXCL9*, and *IFNG* mRNA is used to assess the genetic signature of effector T cells^14^. Our results suggest that the expression of *NLRC3* was significantly correlated with that of *CXCL9* and *CD274* mRNA. There was also a positive correlation found between *NLRC3* and the interferon-γ signaling gene, which is greatly implicated in the clinical response to ICB-based therapy^15^. Notably, patients with high *NLRC3* expression exhibited markedly low TIDE scores, suggesting that patients with LUAD with high *NLRC3* expression can benefit from ICB treatment. The *NLRC3* expression level may therefore predict the ICB response of patients with LUAD. However, this finding needs to be validated in future rodent model studies as well as in clinical practice.

## Conclusions

In LUAD, downregulated *NLRC3* may promote tumorigenesis due to its potential immunomodulatory role. Furthermore, *NLRC3* may be a predictor of patient responses to ICB therapy in the context of LUAD. In summary, NLRC3 could serve as a potential prognostic biomarker for LUAD, help predict the immunotherapeutic response, and guide personalized strategies in the treatment of LUAD.

## Methods

### Data acquisition

RNA sequencing (RNA-seq) data, including data of the cancer group (n = 513) and normal group (n = 59), and the corresponding clinical information of LUAD were retrieved from the TCGA database (https://portal.gdc.cancer.gov/; date downloaded: 08/13/21). The mutation MAF files were downloaded from TCGA database as well (date downloaded: 07/22/22). The gene expression data of normal specimens (n = 578) were obtained from the Genotype-Tissue Expression (GTEx) portal (https://gtexportal.org/home/datasets; latest released version: V8).

### Protein expression analysis

UALCAN (http://ualcan.path.uab.edu/), a website based on the Clinical Proteomic Tumor Analysis Consortium (CPTAC), can be used to analyze the relative protein expression in tumor and normal tissues, as well as the relative protein expression based on individual tumor grade^16^.

### Tumor immune single-cell hub database analysis

The Tumor Immune Single-cell Hub (TISCH, http://tisch.comp-genomics.org/home/) is a scRNA-seq database focusing on the TME^17^. TISCH was used to explore the relationship between *NLRC3* expression and the TME.

### Tumor immune estimation resource database analysis

The Tumor Immune Estimation Resource (version 2.0) (TIMER2.0) database is an online bioinformatics database for estimating the immune infiltration of various cancer types (http://timer.cistrome.org/)^18^. We used the Gene_DE module to analyze differences in gene expression between tumors and normal tissue, the Gene_Outcome module to analyze associations between gene expression and clinical outcomes, and the Gene_Corr module to analyze correlations between genes.

### Prognostic analysis

The 513 analyzed tumor samples were divided into 2 groups: 257 in the high-*NLRC3*-expression group and 256 in the low-*NLRC3*-expression group. Kaplan‒Meier curve survival analyses were used to determine the difference in overall survival (OS) between the high- and low-*NLRC3*-expression groups, which were performed using the “survival” and “survminer” R packages. Univariable and multivariable Cox regression analyses were applied to *NLRC3* expression and seven major clinical factors to identify the appropriate variables for inclusion in the nomogram. A forest plot constructed using the “forestplot” package of R software was used to obtain the HR, 95% CI, and *P* value of each variable. A nomogram was developed based on the results of multivariable Cox proportional hazards analysis to predict the OS probability of each patient. The nomogram was plotted using the “rms” R package.

### Enrichment analysis

Enrichment analysis was performed using Metascape (https://metascape.org/gp/index.html#/main/step1) and WebGestalt (http://www.webgestalt.org/) by inputting the 696 genes that were most positively associated with *NLRC3*^19,20^.

### Evaluation of tumor-infiltrating immune cells

The correlations between the expression levels of *NLRC3* and tumor-infiltrating immune cells were analyzed using the TIMER algorithm implemented in the “ggstatsplot” R package. The proportions of 22 infiltrating immune cells were then inferred using the CIBERSORT algorithm, and the evaluation procedure was performed using the “ggplot2” and “pheatmap” R packages. The correlations among *NLRC3*, chemokines, and chemokine receptor expression were analyzed using the TISIDB database (http://cis.hku.hk/TISIDB/index.php)^21^.

### Immunotherapeutic response predictions

The potential ICB response was predicted using the TIDE algorithm implemented in the “ggplot2” and “ggpubr” R packages^22^.

### Statistical analysis

R software 4.0.3 or 4.1.1 (https://www.r-project.org/) was used in this research. Comparisons between groups were calculated using the Wilcoxon rank-sum test or Kruskal‒Wallis test. The survival curves were generated using the Kaplan‒Meier method and compared statistically using the log-rank test. Survival analyses were performed using the log-rank test and Cox proportional hazards model. The correlation strengths were evaluated using Spearman’s correlation coefficients. Differences with probability values of *P* < 0.05 were considered statistically significant.

### Ethics declaration

This study was conducted in accordance with the Declaration of Helsinki. All procedures were done in accordance with the protocol approved by the Medical Ethics Committee of the First Affiliated Hospital of Xi’an Jiaotong University.

## Data Availability

Publicly available datasets were analyzed in this study. This data can be found here: the data analyzed in this study can be acquired in the TCGA (https://portal.gdc.cancer.gov/), UALCAN (http://ualcan.path.uab.edu/), TISCH (http://tisch.comp-genomics.org/home/), TIMER2.0 (http://timer.cistrome.org/), Metascape (https://metascape.org/gp/index.html#/main/step1), WebGestalt (http://www.webgestalt.org/) and TISIDB (http://cis.hku.hk/TISIDB/index.php) websites.
